# Recent Advances in Gene Therapy for Familial Hypercholesterolemia: An Update Review

**DOI:** 10.3390/jcm11226773

**Published:** 2022-11-16

**Authors:** Qingan Fu, Lijuan Hu, Tianzhou Shen, Renqiang Yang, Long Jiang

**Affiliations:** 1. Department of Cardiovascular Medicine, the Second Affiliated Hospital of Nanchang University, Nanchang 330006, China; 2. Department of Nursing, Nanchang Medical College, Nanchang 330052, China

**Keywords:** familial hypercholesterolemia, antisense oligonucleotide, small interfering RNA, CRISPR/Cas9, gene therapy

## Abstract

(1) Background: Existing lipid-lowering therapies have difficulty in achieving lipid target levels in patients with familial hypercholesterolemia (FH), especially in the treatment of patients with homozygous familial hypercholesterolemia. (2) Method: All of the literature data containing “Familial hypercholesterolemia” and “Gene Therapy” in PubMed and Clinical Trials from 2018 to 2022 were selected. (3) Results: The rapid development of gene therapy technology in recent years is expected to change the treatment status of FH patients. As emerging gene therapy vectors, the optimized adeno-associated viruses, exosomes, and lipid nanoparticles have demonstrated an improved safety and higher transfection efficiency. Various RNA-targeted therapies are in phase 1–3 clinical trials, such as small interfering RNA-based drugs inclisiran, ARO-ANG3, ARO-APOC3, olpasiran, SLN360, and antisense oligonucleotide-based drugs AZD8233, vupanorsen, volanesorsen, IONIS-APO(a)Rx, etc., all of which have demonstrated excellent lipid-lowering effects. With gene editing technologies, such as CRISPR-Cas 9 and meganuclease, completing animal experiments in mice or cynomolgus monkeys and demonstrating lasting lipid-lowering effects, patients with FH are expected to reach a permanent cure in the future. (4) Conclusion: Gene therapy is being widely used for the lipid-lowering treatment of FH patients and has shown excellent therapeutic promise, but the current delivery efficiency, economic burden, immunogenicity and the precision of gene therapy can be further optimized.

## 1. Introduction

Familial hypercholesterolemia (FH) is one of the most common genetic diseases in the world, with the documented prevalence of heterozygous FH (HeFH) and homozygous FH (HoFH), in the population, at about 1/311 and 1/160,000–300,000, respectively [1]. FH is characterized by significantly elevated levels of low-density lipoprotein cholesterol (LDL-C), tendon xanthomas, and premature coronary heart disease (PCHD). There are three major causative genes for FH: low-density lipoprotein receptor (LDLR: accounting for 80–85 % of the factors leading to LDL metabolism disorders), apolipoprotein B100 (apoB100, 5–10%), and proprotein convertase subtilisin/kexin type 9 (PCSK9, 2%) [2]. FH affects tens of millions of people worldwide and brings a heavy economic burden to families and society. Interestingly, the perception of this disease varies greatly in different regions of the world, and there are still many patients in some regions who are not properly diagnosed and treated [3].

The current mainstay of treatment for FH is PCSK9 monoclonal antibody or statin, alone, or combined with ezetimibe. A study reported that the combination treatment with mipomersen resulted in an additional 25% reduction in LDL-C in HoFH patients [4]. Another novel drug, lomitapide, has been shown to reduce LDL-C levels by 50% in patients with HoFH. However, gastrointestinal side effects and potential liver damage limit its clinical use [5]. Lipoprotein apheresis (LA) can reduce LDL-C by more than 65% in patients. However, its potential side effects and expensive costs often make it unaffordable for most patients [6]. Liver transplantation is currently the only way to rapidly manage LDL-C levels of HoFH patients to reach nearly normal, but it is not a routine treatment, due to the significant immune challenges of organ transplantation and the extreme donor shortage [7]. Overall, current therapies have limited therapeutic effects against severe FH, especially HoFH and HeFH, with LDLR-negative mutations.

Since the 21st century, gene therapy has become an important research direction in modern biomedical engineering and clinical disease treatment. Gene therapy embodies the ability to conduct genetic improvements by inserting, removing, or correcting a mutated gene for any targeted therapy. The recent emergence of new technologies, such as small interfering ribonucleic acid (siRNA), antisense oligonucleotides (ASO), clustered regulatory interspaced short tandem repeats (CRISPR), and new transport methods, such as nanomaterials and lipid carriers, have greatly promoted the popularization of gene therapy in clinical practice. Importantly, gene therapy has been used in the clinical treatment of genetic diseases, such as thalassemia, familial cystic fibrosis, RPE65 gene-related retinal diseases, and hemophilia [8]. The advantage of gene therapy lies in the information transmission through genetic mechanisms, which ensure the authenticity, scalability, and stability of the drug delivery and bring lasting therapeutic effects.

**Figure 1:**
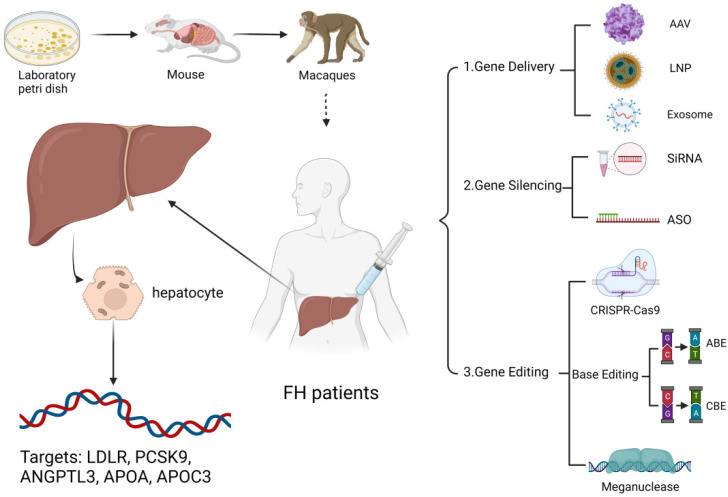
Emerging gene therapy approaches for FH patients. Small interfering RNAs (siRNA) and antisense oligonucleotides (ASO) can inhibit PCSK9 and other targets that are not conducive to lipid-lowering through gene silencing; CRISPER-Cas9, gene editing, and meganuclease can repair dysfunctional LDLR or make LDLR through gene editing. The inactivation of targets that are not conducive to lipid lowering; adenovirus, exosome, and paper nanobody-based delivery tools can deliver the above substances to hepatocytes. (Created with BioRender.com.)

Therefore, much emphasis has been placed on exploring new gene therapies for treating FH patients, especially for HoFH. This review aims to update the progress and challenges of FH-related gene therapy from 2018 (Figure 1).

### Search Strategy

We used the computerized literature databases of PubMed and Clinical Trials, from January 2018 to July 2022. The Medical Subject Heading terms and the following key words were used: “familial hypercholesterolemia”, “gene therapy”, “adeno-associated virus”, “exosome”, “SiRNA”, “antisense oligonucleotides” and “CRISPR/Cas9”. Only English language articles were included in the analysis. In total, 528 articles were retrieved from the database, of which 106 articles were potentially relevant. Among the potentially relevant articles, 50 articles that fit the theme of the review and 14 articles cited from other reviews were included, resulting in a total of 64 articles. Information on all clinical studies included in the review was obtained from the Clinical Trial websites and articles published by the corresponding investigators of the respective projects.

## 2. Replacing a Disease-Causing Gene with a Healthy Copy of the Gene

### 2.1. Adeno-Associated Virus (AAV) Mediated Therapy

The adeno-associated virus (AAV) is a single-stranded linear DNA-deficient virus with a genome of 4.7 Kb and is well-recognized as an excellent gene therapy vector because it hardly induces any immune response, has a low risk of integration into the host genome and can permanently express the therapeutic genes, compared with other viral vectors [9]. AAV-mediated gene therapy is reportedly a successful approach for treating monogenic diseases. In recent years, AAV-mediated targeted hepatocyte gene therapy has shown excellent efficacy and durable duration of action in clinical trials in patients with hemophilia [10]. Similarly, AAV-mediated therapy has shown promising results in pre-clinical trials of Duchenne muscular dystrophy (DMD) [11].

A phase 1/2 clinical trial (NCT 02651675) was completed in 2020 (Table 1) to evaluate the safety and efficacy of the AAV vectors in nine HoFH patients. The intravenous administration of the thyroxin-binding globulin (TBG) promoter directed to control the AAV enhanced liver-specific LDLR expression (AAV8.TBG.hLDLR) and reduced the LDL-C level in the peripheral blood [12]. Although the results have not been published, preliminary findings indicated asymptomatic elevations of the liver transaminases in the participants, possibly due to the T cell immune responses to the carrier capsid [13].

To improve the efficacy of the AAV vectors, researchers developed new AAV vectors which combine AAV8 with the intervening sequence 2 (AAV8-IVS2) of the human beta-globin gene and injected with a single dose of AAV8-IVS2 plus LDLR (hLDLR011-T) in 11 week old Ldlr−/−, Apobec1−/− double-knockout (DKO) mice with a chow diet [14]. The results showed a 99% reduction of the serum LDL-C levels on day three, which persisted until day 120. Overall, the new generation vector AAV8-IVS2 had a higher transduction efficiency with an ideal efficacy at a safe dose, avoiding side effects, suggesting it has huge potential for application in the treatment of HoFH patients.

Although AAV is a promising gene therapy vector, it still faces significant challenges before a large-scale clinical application (Table 2). First, the transfection efficiency of the AAV vector into human hepatocytes is lower than that of mouse hepatocytes, resulting in a weakened lipid-lowering effect in humans, emphasizing the need to further optimize the transfection efficiency of the vector. Although no obvious side effects were observed in the AAV-mediated gene therapy, human lipid metabolism is complex, and long-term treatment may lead to changes in the lipid profiles or abnormal fat accumulation. Accordingly, long-term follow-up studies are warranted to ensure treatment safety [15]. In addition, the turnover of hepatocytes in children is rapid when treating FH. The efficacy of intrahepatocellular AAV vectors in vivo may be more transient over time, and increasing the frequency of treatment may achieve stable and long-lasting therapeutic effects in vivo [16].

### 2.2. Exosome-Mediated Therapy

An exosome is a disk-shaped vesicle originating from the endosome of the nucleus, with a density of 1.13–1.18 g/mL and a diameter of 40–160 nm [17]. As “natural nanoparticles”, exosomes are very stable and can be loaded with mRNA, miRNA, protein, and plasmid DNA, to cross the various biofilm barriers. Compared with artificial nanoparticles, exosomes avoid the rapid recognition and clearance of phagocytes in vivo and have a good biocompatibility [18]. Studies have begun to focus on the therapeutic role of exosomes in specific diseases, such as delivering IL-10 mRNA to control inflammation in patients with atherosclerosis [19], targeting STAT3 for the treatment of liver fibrosis [20], repairing myocardial injury and treating malignant tumors [21,22].

Recently, researchers documented an exosome-based Ldlr gene therapy for FH in an Ldlr−/− mouse model. First, they transferred the Ldlr encoding sequence (CDS) downstream of the plasmid’s EF1α promoter and fused it with the marmot hepatitis virus post-transcriptional regulatory element (WPRE). Then, the vector was transfected into packaging cells to enrich the LDLR in exosomes (ExoLdlr). Following the injection of ExoLdlr into Ldlr−/− mice, fed a high-fat diet for eight weeks, the expression of the LDLR protein was significantly increased in the liver of the ExoLdlr mice and the level of LDL-C in the peripheral blood was significantly decreased (from 6.5 mmol/L to 4.5 mmol/L). In addition, after eight weeks of the injection of ExoLdlr, the fatty liver and atherosclerosis were improved [The atherosclerotic area of ExoLdlr mice (3%) was significantly lower than that of the PBS group (15%)] in mice fed a high-fat diet for 16 weeks [23]. The HE staining of mice organs showed that the exosome treatment had no obvious toxicity or safety issues.

However, some aspects of the exosome therapy need to be strengthened before clinical translation. For instance, it is essential to increase the production of high-purity exosomes to prevent off-target effects, to transfer large RNAs, and quantify the load of each exosome. The first human study of exosome-based LDL mRNA delivery began in December 2021, involving 30 patients diagnosed with HoFH, in a phase I clinical trial (NCT05043181). The researchers plan to inject exosomes into the patient’s intraperitoneal cavity under ultrasound guidance to observe the changes in the patient’s peripheral blood LDL level, and we look forward to the publication of the results of this trial.

## 3. Inactivating a Disease-Causing Gene That Is Not Functioning Properly

### 3.1. Small Interfering RNA (SiRNA)

SiRNA is a type of double-stranded RNA with a length of 20–25 nucleotides, mainly involved in the RNA interference (RNAi) phenomena in vivo. SiRNA-based therapies are reportedly effective in rare diseases, such as hereditary transthyretin amyloidosis, transthyretin amyloidosis, hepatic transthyretin amyloidosis, transthyretin amyloidosis, porphyria, and type 1 hyperoxaluria [24,25,26].

Inclisiran (ALN-PCSsc) is a long-acting synthetic double-stranded siRNA that directly targets PCSK9 mRNA, by specifically binding to the glucose-lowering glycoprotein receptor (ASGPR) and the triantennary N-acetylgalactosamine carbohydrate (GalNAc) ligand [27]. PCSK9 is a key factor in the lipid regulation, which can inhibit the expression of LDLR in the liver and thereby increase the level of LDL-C. The PCSK9 LOF mutations reduce the circulating LDL-C level and ultimately further reduce the risk of CHD [28]. In a randomized controlled of a double-blind phase 3 trial (NCT03400800), patients with atherosclerosis were randomized 1:1 to receive inclisiran (284 mg) or a matching placebo, via 1.5 mL subcutaneous injections. On day 510, the incidence of a 50% reduction in LDL-C in the inclisiran group was significantly higher than in the placebo group (61.5% vs. 2.2%) [29]. On 9 December 2020, inclisiran received the European Union’s approval for primary hypercholesterolemia or mixed dyslipidemia [30] and would be clinically combined with conventional lipid-lowering drugs. Inclisiran is well tolerated, except for mild to moderate bronchitis and mild skin reactions at the injection site [29]. A multicenter, non-interventional, non-randomized, and prospective cohort study (NCT05362903) is underway to observe the lipid-lowering effects and the safety of inclisiran in different types of patients (non-medication patients, oral statin patients, and regular lipid apheresis patients).

ARO-ANG3 is a siRNA-based inhibitor of angiotensin-like protein 3 (ANGPTL3). ANGPTL3 is a protein produced by the liver that regulates lipoproteins by inhibiting the function of the endogenous lipase [31]. In one study, wild-type mice injected with a siRNA drug targeting ANGPTL3 showed a 95% decrease in the liver ANGPTL3 expression and a decrease in the serum LDL-C (−40%) and HDL-C (−50%) on day five, compared with the controls. The researchers injected obese mice with the same targeted drug and observed a significant reduction in the ANGPTL3 levels on day three (about 97%) and a 56% reduction on day 24. Plasma LDL-C levels decreased by 73% and 84% on day three and day 10, respectively [32]. In addition, the pharmacokinetics and safety of a siRNA-based approach to the ANGPTL3 inhibition in dyslipidemia and healthy volunteers were assessed in a phase 1 study (NCT03747224). The preliminary results showed a 42% reduction in LDL-C. In another published phase 1/2a single-dose-ranging study, ARO-ANG3 reduced ANGPTL3 by 43–75% and triglycerides (TG) by 47–53%, with no serious adverse reactions [33]. A phase II trial (NCT04832971) of 204 adults with mixed dyslipidemia is underway to evaluate the efficacy and safety of ARO-ANG3.

ARO-APOC3 is a siRNA drug targeting APOCIII. APOCIII is a 79-amino acid glycoprotein which is synthesized primarily in the liver. APOCIII regulates the triglyceride levels mainly by inhibiting the hepatic clearance of the triglyceride-rich lipoproteins (TRLs) and increasing the very low density lipoprotein (VLDL) [34]. A LOF mutation in APOC3 has been shown to reduce the TG levels by 40% and reduce the risk of coronary heart disease (CHD) by 40% [35]. In a recent phase 1/2 study, 40 healthy volunteers with fasting TG > 80 mg/dL were divided into treatment and placebo groups. Then, 16 weeks later, the LDL-C decreased by 42 to 53% in the treatment group, while the TG decreased by 41–55%, and no serious adverse events occurred [36]. A single-dose and multidose escalation phase 1 study (NCT03783377) involving 112 patients with fasting serum triglycerides of at least 300 mg/dL (3.38 mmol/L) ended on 11 February 2021, but the results have not yet been published.

Lipoprotein(a) [Lp(a)] is a LDLC–like particle bound to apolipoprotein(a). Lp(a) is an independent risk factor for (ASCVD), which affects the prognosis of FH patients and is also a potential therapeutic target [37]. In the FOURIER trial results, it was observed that the PCSK9 inhibitors could reduce the plasma Lp(a) level, and the risk of cardiovascular events was reduced by 20% after a 34 nmol/L absolute reduction in Lp(a) [38]. A mendelian randomization analysis showed that reducing Lp(a) by 65–100 mg/dL, reduced the risk of CHD by the equivalent of a 38 mg/dL reduction in LDL-C levels [39]. There are two siRNA drugs for lowering Lp(a) levels. One is olpasiran (AMG 890) which targets Apo(a) mRNA. In an animal study, after a single subcutaneous injection of 3 mg/kg and 10 mg/kg, olpasiran in cynomolgus monkeys, the serum Lp(a) concentrations remained >80% lower than the baseline on day 45 after reaching their nadir. For the 2 mg/kg olpasiran group, the results showed that the plasma Lp(a) level of the cynomolgus monkeys reached a nadir (>90% reduction) on day 22 [40]. In a phase 1 clinical trial (NCT03626662), which included 48 patients with Lp(a) ≥ 70 nM, the largest reduction in Lp(a) was observed between days 43 and 71; Lp(a) levels decreased by 76% to 91% from the baseline in patients with Lp(a) ≥ 200 nM, and decreased by 71% to 97% in patients with 70 nM ≤ Lp(a) ≤ 199 nM [37]. No serious adverse events occurred in this study. Another phase 2 clinical study (NCT04270760), which includes 240 patients with Lp(a) levels ≥ 150 nmol/L and ASCVD, is currently underway to determine the decline in the Lp(a) levels in patients at week 36 and to evaluate the benefits of different subcutaneous injections. Another siRNA for Lp(a) is SLN360, a 19-mer siRNA (conjugated GalNAc) drug. In a phase 1 clinical study (NCT04606602), investigators administered SLN360 via an abdominal subcutaneous injection to 32 participants with Lp(a) levels greater than 150 nmol/L and no known cardiovascular disease. Participants receiving 300 mg and 600 mg of SLN360, experienced a 70% and 80% reduction in Lp(a) concentrations from the baseline at day 150, respectively, and the largest reductions in LDL-C and TC levels were 26% and 18%, respectively. The levels of Lp(a) in the 300 mg group reached a nadir on day 45 (96%), and the maximum reduction of Lp(a) in the 600 mg group was 98% (day 60) [41]. Overall, the siRNA-based methods for lowering Lp(a) have huge prospects for clinical application, but the current understanding was based on a limited number of participants. Accordingly, we still need larger-scale clinical studies to further understand the efficacy and safety of the drug.

### 3.2. Antisense Oligonucleotides (ASO)

Antisense oligonucleotides (ASO) are short (16–53 nt) single-stranded nucleic acid sequences, usually highly complementary to small RNAs, that prevent protein translation by degrading mRNA or by competitive inhibition [42].

ASO plays an important role in gene therapy, but naked ASO cannot penetrate negatively charged cell membranes to reach the interior of the cells. Thus, the complexity of the ASO delivery remains an important challenge for the ASO-based therapy. Nonetheless, lipid nanoparticles (LNPs) provide a good way for its delivery. In one study, a synthetic lipid 306-O12B-3 was selected from a lipid library to form LNPs for the in vivo delivery of PCSK9-ASO. The experimental mice were injected with 0.05, 0.1, 0.5, 1, and 1.5 mg/kg^−1^ PCSK9-ASO/LNP complexes. Then, 72 h later, the liver tissue sections were quantitatively detected by RT-PCR, and the results showed a dose-dependent decrease of PCSK9 mRNA. The PCSK9 mRNA expression of mice injected with 1.5 mg/kg^−1^ decreased to 25% of the initial levels, and the corresponding PCSK9 protein and serum cholesterol levels also decreased significantly [43].

It is widely acknowledged that the currently available PCSK9 inhibitor needs to be administered by injection [44], while the recently proposed AZD8233 (ION-863633) can be administered orally, which undoubtedly provides a more convenient alternative for patients to take the drug. AZD8233 is a GalNAc ASO targeting PCSK9 mRNA with a restricted ethyl (cEt) chemistry. A study (NCT03593785) in a population with a baseline LDL cholesterol of 134 ± 3.9 mg/dl found a dose-dependent reduction in human plasma PCSK9 protein concentrations at single doses of 12, 30, and 90 mg of AZD8233. Plasma PCSK9 levels were reduced by up to 95%, with a corresponding LDL-c reduction of up to 68% with a single dose of 90 mg [45]. The results of this study confirmed the high efficacy of the drug. Indeed, a single dose of 90 mg was sufficient to induce a >90% reduction in circulating PCSK9, compared to the monoclonal antibodies and yielded a better performance than the siRNA-type drugs (80% reduction), providing a new option for patients treated with traditional PCSK9 inhibitors [46].

IONIS-ANGPTL3-Lrx (vupanorsen) is an ANGPTL3 inhibitor of the conjugated ASO. In a phase II study (NCT03371355), the mean triglyceride reductions of 47%, 36%, and 53% were observed with injections of 20 mg, 40 mg, and 80 mg of vupanorsen once a week in a four-week period, respectively [47]. However, due to the poor lipid-lowering effect and the dose-dependent fatty liver, Pfizer recently announced they were discontinuing the development of vupanorsen.

The first drug to target ApoC3 mRNA was volanesorsen, and other drugs that inhibit ApoC3 are still in the early stages of research. The APPROACH trial (NCT02211209) was a Phase 3 trial that studied the efficacy and safety of volanesorsen in patients with familial chylomicronemia syndrome (FCS). The experiment included 66 FCS patients, 1:1 divided into experimental and control groups. The experimental group received 300 mg of volanesorsen every week [48]. Three months later, the study found that patients in the experimental group experienced an 84% reduction in APOC3 levels and a 77% reduction in mean plasma TG levels, compared with a 6.1% increase in APOC3 and 18% increase in TG in the placebo group. Although experiments have substantiated the effect of volanesorsen in lowering lipids, the high incidence of concomitant thrombocytopenia restricts its clinical application. Olezarsen (AKCEA-APOCIII-LRx) may replace volanesorsen in the future since it contains the antisense oligonucleotides of apoC3 and GalNac. It has a stronger APOC3 antagonism and a higher hepatic first-pass clearance. Most importantly, no case of thrombocytopenia was reported. In a phase 1/2a trial of 67 healthy volunteers with mildly elevated triglyceride levels (NCT02900027), AKCEA-APOCIII-LRx reduced apoC3 (−92%), and triglyceride (−77%) levels dramatically dropped [49]. In a closed phase 2 dose-ranging trial (NCT03385239), AKCEA-APOCIII-LRx was used in 114 participants with established ASCVD or at high cardiovascular risk and triglyceride levels > 200 mg/dL. The trial aimed to explore the drug’s efficacy in treating cardiovascular disease patients and improving their prognosis, but the results have not yet been published.

The first-generation ASO drug IONIS-APO(a)Rx targets apo(a) mRNA, providing a direct pathway to inhibit the Lp(a) mRNA expression. Although IONIS-APO(a)Rx reduces Lp(a), its high frequency of injection and high-dose administration is a problem for patients [50]. The second-generation drug IONIS-APO(a)Rx-lrx, conjugated with GalNAc, reportedly overcomes this problem with an improved potency and tolerability without adverse reactions. Its efficacy was confirmed in a phase 2 study (NCT03070782) involving 286 patients with confirmed CVD with Lp(a) > 150 nmol/L. Results showed a dose-dependent decrease in the Lp(a) levels, with an 80% reduction at 20 mg per week, 58% at 20 mg every two weeks, and 35%, 56%, and 72% at 20, 40, and 60 mg every four weeks, respectively [51]. The ongoing phase 3 study of Lp(a)HORIZON (NCT04023552) will provide additional clinical evidence of the drug’s safety and efficacy in reducing the ASCVD risk [52].

## 4. Introducing a New or Modified Gene into the Body for Therapy

### 4.1. CRISPR-Cas 9

The CRISPR/Cas9 system is the third generation of gene editing tool after ZFNs and TALENs. The gene editing of cells and organisms using CRISPR/Cas9 is more efficient and precise than previous technologies, in addition to being simpler and easier to fabricate [53]. Accordingly, the CRISPR/Cas9 system is currently the genome editing tool of choice [9], and the development of this technology was recognized by the Nobel Prize Committee in 2020.

In a recent study, lipid nanoparticles containing the CRISPR base editor were injected into cynomolgus monkeys. The results showed that the blood levels of LDL-C and PCSK9 were significantly reduced by 60% and 90%, respectively, and PCSK9 was almost completely cleared from the liver. This effect was sustained for eight months after a single injection dose [54]. Researchers used the AAV-CRISPR/Cas9 system in another study to gene-edit a newly constructed LdlrE208X mutant mouse model [12]. The levels of LDL-C (−66.8%), TC (−68.5%) and TG (−55.6%) in the peripheral blood of mice were significantly decreased after editing, and the area of atherosclerotic plaque was also significantly decreased (−61.7%).

In 2021, based on the CRISPR-CAS9 system, researchers used 306-O12B LNP to edit and knock down ANGPTL3 in C57BL/6 mice. Dose-dependent reductions in ANGPTL3(−60%), TG (−28.6%), and LDL-C (−48.1%) levels were still observed 100 days after a single dose (3.0 mg/kg) [55]. Another study used CRISPR-Cas9 gene editing technology to induce the permanent LOF mutation in ANGPLT and found that the TC, TG and ANGPTL3 levels were reduced by 19%, 31%, and 49%, respectively, after seven days of the injection in five-week-old male wild-type mice. In the five-week-old hyperlipidemic Ldlr-knockout mice, the TC and TG levels were reduced by 51%, and 56% in the ANGPLT mutated mice [56,57]. The above results suggest that this approach may be a boon for treating HoFH patients.

CRISPR-cas9 technology can also be combined with the induced pluripotent stem cells (iPSCs) to restore the function of LDLR to regulate the lipid metabolism. iPSCs can be differentiated into hepatocyte-like cells (HLCs), and iPSC-derived HLCs can serve as models for FH studies. Researchers obtained iPSC-derived HLCs (HoFH-iPSCs) of lymphocytes from a HoFH patient and wild-type iPSCs (WT-iPSCs) from healthy volunteers. Genome sequencing revealed that HoFH-iPSCs share the same LDLR mutation and lipid metabolism disorder as HoFH patients. Homozygous gene-corrected (gcHoFH+/+−iPSCs) and heterozygous gene-corrected (gcHoFH+/−−iPSCs) were obtained after the CRISPR-cas9 modification. The results showed that the uptake capacity of LDL in gcHoFH+/+−HLCs (43.7%) was close to that of WT-HLCs (49.6%) and higher than gcHoFH+/−−HLCs (28.3%) and HoFH-HLCs (18.0%) [58]. This result confirms that CRISPR-cas9 technology can be used to restore the LDLR function in HoFH patients. In addition, researchers observed little immune response in vitro of the patient’s lymphocytes to the autologous iPSC-derived HLC (genetically modified). There are still some limitations in this study. First, the ability of gcHoFH-HLCs to take up LDL was not compared with that of human hepatocytes. Furthermore, whether gene correction in such cells might lead to insertional tumorigenesis was not investigated. Overall, the iPSCs-based CRISPR-cas9 has a great research potential and may be able to fundamentally treat HoFH with little concern for immune responses.

The CRISPR/Cas9 gene editing system is undoubtedly a breakthrough technology, bringing hope for improvement, especially for those patients with severe HoFH. Despite the great potential of CRISPR/Cas9 technology, some safety and ethical issues limit its clinical application. First, the double-strand breaks caused by the introduction of CRISPR/Cas9 technology, the lack of sufficient specific sgRNA, and the repair of random insertions and deletions may lead to unpredictable off-target mutations in the target genome, thereby increasing the risk of clinical treatment. Furthermore, after selecting an appropriate gene to correct and repair, there may still be a potential risk of developing tumors. Moreover, CRISPR/Cas9 also has ethical limitations, especially when editing human germ cells, which must raise ethical considerations. With further technological progress, CRISPR/Cas9 can be used to edit individual mutations in differentiated human cells, minimizing the chance of off-target effects and accidental point mutations [59].

### 4.2. Base Editing (BE)

Another similar technique is in vivo base editing (BE). A nonsense variant of PCSK9 was introduced using a base editor (BE3), packaged in an adenovirus, and injected into eight-week-old mice. The results showed that base editing of Pcsk9 resulted in a 56% and 28% reduction in plasma PCSK9 protein levels and plasma cholesterol levels, respectively, slightly lower than the levels observed after the modification with CRISPR-Cas9 [60]. In vivo base editing was performed in five-week-old male C57BL/6J mice by injection of BE3-ANGPTL3, and a significant decrease in the plasma levels of ANGPTL3, cholesterol, and triglycerides was observed after seven days (49%, 19%, and 31%, respectively) [56]. In addition, editing using BE3 is more precise, with almost no chromosomal translocation or off-target editing and can solve the adverse effects of CRISPR-Cas9, leading to a lower probability of the immune response [61].

### 4.3. Meganuclease

Homing endonuclease (Meganuclease) is a deoxyribonuclease that can recognize longer DNA sequences (14–40 bp) and can be used for gene editing. Interestingly, the iCREI-derived megacurases from Chlamydomonas rheinistica are more difficult to make than other editing tools, such as CRISPR-Cas9, but their higher specificity and small size (about 300 amino acids) make them a promising gene editing method [62].

In non-human primates (NHPs), Wang et al. demonstrated that AAV-delivered meganuclease knockout PCSK9 could reduce plasma LDL levels. Combining engineered meganucleases with AAV8, Wang et al. made M1PCSK9 (first generation) and M2PCSK9 (second generation), targeting the PCSK9 gene exon 7 sequence conserved between humans and macaques. Following injection of AAV8-M1PCSK9, all four rhesus monkeys exhibited the dose-dependent inactivation of PCSK9 within six weeks and decreased serum cholesterol for up to 11 months. In two rhesus monkeys, serum LDL was reduced by 34% after injection of (6.0 × 1012 GC/kg) AAV8-M2PCSK9. In addition, the results showed that, compared with the first generation, the second generation AAV8-M2PCSK9 has a lower off-target rate and higher identification accuracy [63]. The continuous monitoring for three years showed stable low serum PCSK9 and LDL-C levels, with no obvious adverse reactions [64].

It is widely thought that meganuclease-mediated gene editing may have a therapeutic effect in some FH patients, but also yields some problems. First, M2PCSK9 may have a lower off-target rate, the risk of the off-target cleavage of the vector genome remains unclear. Moreover, there was a slight increase in transaminases in the experiment. Although no safety issues were observed, the accuracy of these findings may have been affected to a certain extent by the small sample size and a short follow-up time. Further research is needed to reduce its off-target probability and potential immunotoxicity before clinical trials.

## 5. Conclusions and Perspectives

Due to the potentially high risk of cardiovascular events in FH, the effective treatment and management of this patient population have been a daunting challenge for the global medical community. Since the 21st century, with the development of gene therapy technology, HoFH patients have seen the hope of a cure. The breakthrough from the traditional gene replacement to gene editing has brought infinite possibilities for treating FH. In addition to the gene editing systems, such as CRISPR/Cas9, new technologies, such as nanomaterials and IPSC have also shown great promise in basic and early clinical research. With further development of technology, gene therapy may allow FH patients to obtain permanent benefits from a single therapy safely and ethically, thereby fundamentally reducing the global economic burden associated with FH.

## Figures and Tables

**Table 1:** Clinical trials for gene therapies.

Way.	Target	Drug Name	Study Design	Sample Size	Primary Outcome	Lipid-Lowering Effect	Trial Number
**AAV-mediated therapy**	LDLR	AAV8.TBG.hLDLR	Phase 1/2	9 participants	Number of participants with product-related adverse events, up to 24 weeks	Not shown	NCT02651675
**Exosome-mediated therapy**	LDLR	ExoLdlr	Phase 1	30 participants	Changes of TC, TG, LDL-C, HDL-C at day 19	Underway	NCT05043181
**SiRNA**	PCSK9	Inclisiran (ALN-PCSsc)	Phase 3	1617 participants	Percentage change in LDL-C from the baseline to day 510	LDL-C reduction 50%	NCT03400800
	ANGPTL3	ARO-ANG3	Phase 1	93 participants	Number of participants with treatment-related adverse events, post-dose 113 (±3 days)	LDL-C reduction 42%	NCT03747224
			Phase 2	204 participants	Percentage change in fasting TG from the baseline, at week 24	Underway	NCT04832971
	APOC3	ARO-APOC3	Phase 1	112 participants	Number of participants with possible treatment-related adverse events as of day 113 (±3 days)	Not shown	NCT03783377
	Lp(a)	Olpasiran (AMG 890)	Phase 1	79 participants	Changes in the data, such as participant incidence of treatment adverse events and Lp(a) levels, up to 365 days	Lp(a) reduction 76–91% in patients with Lp(a) ≥ 200 nM/L, 71% to 97% in patients with 70 nM/L ≤ Lp(a) ≤ 199 nM/L	NCT03626662
			Phase 2	290 participants	Percentage change in Lp(a), relative to the baseline values, at week 36	Underway	NCT04270760
		SLN360	Phase 1	88 participants	Safety and tolerability of SLN360 in patients with elevated Lp(a)	Lp(a) reduction 70% (300 mg) or 80% (600 mg)	NCT04606602
**ASO**	PCSK9	AZD8233 (ION-863633)	Phase 1	73 participants	Safety, tolerability, and pharmacokinetics of AZD8233, from the randomization to 4 months follow-up	PCSK9 reduction 95%, LDL-C reduction 68%	NCT03593785
	ANGPTL3	Vupanorsen(IONIS-ANGPTL3-Lrx)	Phase 2	105 participants	Percentage change from the baseline in the fasting TG levels, week 25 for cohorts B and C, week 27 for cohort A	TC was reduced by 47%, 36% and 53%, respectively, with weekly injections of 20 mg, 40 mg and 80 mg vupanorsen for four weeks.	NCT03371355
	ApoC3	volanesorsen	Phase 3	67 participants	Fasting TG relative to the baseline percentage change, at month 3	ApoC3 reduction 84%; TG reduction 77%	NCT02211209
	ApoC3	Olezarsen (AKCEA-APOCIII-LRx)	Phase 1/2a	56 participants	Safety, tolerability, pharmacokinetics, and pharmacodynamics of IONIS APOC-III-LRx, up to 183 days	ApoC3 reduction 92%; TG reduction 77%	NCT02900027
			Phase 2	114 participants	Safety, tolerability and effectiveness of ISIS 678354 in reducing the TG levels at different doses, up to 6 months	Underway	NCT03385239
	Lp(a)	ISIS 681257 (IONIS-APO(a)Rx-lrx)	Phase 2	286 participants	Percentage change from the baseline in Lp(a), week 25 for groups A, B, and C, week 27 for groups D and E	Lp(a) reduction 80% (20 mg per week), 58% (20 mg per 2 weeks), 35%, 56%, 72% (20, 40, 60 mg per 4 weeks)	NCT03070782
		TQJ230	Phase 3	8324 participants	Time to first adverse cardiovascular event in the group of patients with elevated Lp(a) ≥ 70 or 90 mg/dL, time frame: approximately 4 years	Underway	NCT04023552

**Table 2:** The advantages and challenges of the different approaches.

Approach	Advantage	Challenge
**AAV-mediated therapy**	Higher transduction efficiency. Better efficiency at safe doses. Fewer side effects.	The transfection efficiency of the AAV vector into human hepatocytes is lower than that of mouse hepatocytes. Long-term treatment may lead to changes in the lipid profiles or abnormal fat accumulation. Higher production cost.
**Exosome-mediated therapy**	Very stable. Avoid the rapid recognition and clearance of phagocytes in vivo. Have a good biocompatibility.	Low yield of high-purity exosomes. Poor ability to transfer large RNAs. Load of each exosome needs to be quantified.
**RNA-targeted therapeutics** **(SiRNA, ASO)**	Higher specificity for the target. Lower potential for off-target adverse effects. Lower frequency of administration.	It is easily degraded in vivo. The off-target rate needs to be further reduced.
**CRISPR-Cas 9**	More efficient and precise. Simpler and easier to fabricate.	Unpredictable off-target mutations in the target genome. Following the selection of an appropriate gene to correct and repair, there may still be a potential risk of developing tumors. CRISPR/Cas9 also has ethical limitations.
**Base editing**	More precise. Almost no chromosomal translocation or off-target editing and can solve the adverse effects of CRISPR-Cas9. Lower probability of immune response.	The operation efficiency is low. The scope of application is not broad enough.
**Meganuclease**	Higher specificity. Smaller size (about 300 amino acids).	Harder to make than other editing tools, such as CRISPR-Cas9. The risk of off-target cleavage of the vector genome remains unclear. There was a slight increase in transaminases in the experiment.

## Data Availability

Not applicable.

## Abbreviations

AAV.	Adeno-associated virus
ANGPTL3	Angiotensin-like protein 3
ASGPR	Glucose-lowering glycoprotein receptor
ASO	Antisense oligonucleotides
ASCVD	Atherosclerotic cardiovascular disease
BE	Base editing
CDS	Encoding sequence
CHD	Coronary heart disease
DKO	Double-knockout
FCS	Familial Chylomicronemia Syndrome
FH	Familial hypercholesterolemia
GalNAc	N-acetylgalactosamine carbohydrate
HeFH	Heterozygous familial hypercholesterolemia
HFD	High-fat diet
HLCs	Hepatocyte-like cells
HoFH	Homozygous familial hypercholesterolemia
iPSCs	Induced pluripotent stem cells
LDL-C	Low-density lipoprotein cholesterol
LDLR	Low-density lipoprotein receptor
Lp(a)	Lipoprotein(a)
LOF	Loss of function
NHPs	Non-human primates
PCHD	Premature coronary heart disease
PCSK9	Proprotein convertase subtilisin/kexin type 9
RNAi	RNA interference
siRNA	Small interfering ribonucleic acid
TBG	Thyroxin-binding globulin
TG	Triglycerides
TC	Total cholesterol
TRLs	Triglyceride-rich lipoproteins
VLDL	Very low density lipoprotein
WPRE	Marmot hepatitis virus post-transcriptional regulatory element
